# Brucellosis in mammals of Costa Rica: An epidemiological survey

**DOI:** 10.1371/journal.pone.0182644

**Published:** 2017-08-09

**Authors:** Gabriela Hernández-Mora, Roberto Bonilla-Montoya, Osvaldo Barrantes-Granados, Andrea Esquivel-Suárez, Danilo Montero-Caballero, Rocío González-Barrientos, Zeanne Fallas-Monge, José David Palacios-Alfaro, Mario Baldi, Elena Campos, Grettel Chanto, Elías Barquero-Calvo, Carlos Chacón-Díaz, Esteban Chaves-Olarte, Caterina Guzmán Verri, Juan-José Romero-Zúñiga, Edgardo Moreno

**Affiliations:** 1 Servicio Nacional de Salud Animal (SENASA), Ministerio de Agricultura y Ganadería, Heredia, Costa Rica; 2 Instituto Nacional de Aprendizaje, San José, Costa Rica; 3 Fundación Keto, San José, Costa Rica; 4 Programa de Investigación en Enfermedades Tropicales (PIET), Escuela de Medicina Veterinaria, Universidad Nacional, Heredia, Costa Rica; 5 Centro Nacional de Referencia en Bacteriología, Instituto Costarricense de Investigación y Enseñanza en Nutrición y Salud (INCIENSA), Cartago, CR; 6 Facultad de Microbiología, Centro de Investigación en Enfermedades Tropicales (CIET), Universidad de Costa Rica, San José, Costa Rica; 7 Programa de Investigación en Medicina Poblacional, Escuela de Medicina Veterinaria, Universidad Nacional, Heredia, Costa Rica; 8 Instituto Clodomiro Picado, Universidad de Costa Rica, San José, Costa Rica; Institut National de la Recherche Agronomique, FRANCE

## Abstract

Brucellosis has been an endemic disease of cattle and humans in Costa Rica since the beginning of XX century. However, brucellosis in sheep, goats, pigs, water buffaloes, horses and cetaceans, has not been reported in the country. We have performed a brucellosis survey in these host mammal species, from 1999–2016. In addition, we have documented the number of human brucellosis reported cases, from 2003–2016. The brucellosis seroprevalence in goat and sheep herds was 0.98% and 0.7% respectively, with no *Brucella* isolation. Antibodies against *Brucella* were not detected in feral or domestic pigs. Likewise, brucellosis seroprevalence in horse and water buffalo farms was estimated in 6.5% and 21.7%, respectively, with no *Brucella* isolation. Six cetacean species showed positive reactions against *Brucella* antigens, and *B*. *ceti* was isolated in 70% (n = 29) of striped dolphins (*Stenella coeruleoalba*). A steady increase in the diagnosis of human brucellosis cases was observed. Taking into account the prevalence of brucellosis in the various host mammals of Costa Rica, different measures are recommended.

## Introduction

Costa Rica (CR) is a Central American country with a surface area of 51100 Km^2^ and a human population close to five million. Most of the inhabitants are located in the Central Valley, flanked by the volcanic chain and the mountain range. The country is divided in six administrative areas: Chorotega, Central Pacific, Brunca, Central, Northern Huetar and Caribbean Huetar. CR has two ocean fronts: the Pacific Ocean and the Caribbean Sea. In addition, there is the Cocos Island located in the Pacific Ocean [1].

Bovine brucellosis is a significant problem in CR [2] and human brucellosis has been endemic since the beginning of last century [3,4]. However, the presence of *Brucella* organisms in sheep, goats, pigs, water buffaloes, horses and cetaceans and the impact that brucellosis has in these animals has been barely explored in CR [5]. Moreover, very little information in the number of human cases arriving to the CR health centers has been recorded.

Up to now, five species of *Brucella* have been isolated in CR: *Brucella abortus* (biotypes 1, 2 and 3) in cattle and humans, *Brucella suis* (biotype 1) in domestic swine, *Brucella canis* in dogs, *Brucella neotomae* in humans and *Brucella ceti* (dolphin type) in dolphins [2,5–7]. *B*. *melitensis* and *B*. *ovis* have not been reported in CR.

In this work we describe the distribution and the prevalence of brucellosis in different mammal species and the cumulative number of human brucellosis cases in CR. We discuss our findings in concordance to the conditions and measures carried out in the country and the zoonotic potential. Brucellosis in cattle is not reported here, since it has been thoroughly described in the accompanying manuscript [2].

## Materials and methods

### Serum samples

#### Sheep and goats

The total number of sheep and goats in CR is close to 12358 and 4626, distributed in about 164 and 271 herds, respectively (Table 1). For sampling purposes CR was divided in six administrative areas by the Costa Rican National Animal Health Service (CR-NAHS) of the Ministry of Agriculture and Livestock Management: Chorotega, Central Pacific, Brunca, Central, Northern Huetar and Caribbean Huetar. Herds from each species were divided in three sections. For sheep the first section “A” included 6200 animals in 22 herds of broodstock farms with ≥150 individuals; section “B” were 3577 animals in 37 herds from farms with eventual broodstock activities, with populations ranging from 149–60 animals; and section “C” were 2691 in 105 herds for productive farms with population ≤59 animals. For goats, we used the same criteria used for sheep. Section “A” included 1406 goats in 13 herds; section “B” were 1603 distributed in 14 herds; and section “C” were 1617 from 137 farms. Seventy-eight caprine and 139 ovine herds, corresponding to 2013 and 1668 animals respectively, were sampled nationwide as part of the surveillance program, during 2014–2016.

**Table 1:** Numbers of ovine and caprine herds and numbers of animals by geographical region in Costa Rica (2015).

Region	Ovine		Caprine	
	Herd	Animals	Herd	Animals
**1. Northern Huetar**	36	2440	39	2077
**2. Central**	59	4295	117	1973
**3. Brunca**	21	1246	41	128
**4. Chorotega**	28	2792	22	312
**5. Caribbean Huetar**	9	637	28	79
**6. Central Pacific**	11	948	24	57
**Total**	**164**	**12358**	**271**	**4626**

#### Water buffalos

The estimated water buffalo population in the country corresponds to 13000 animals, distributed in about 100 herds. About 70% of the water buffalo farms are devoted to mozzarella cheese production. The rest, are dedicated to meat production, leather industry or as wild fauna in zoological parks [8,9]. A total of 2586 animal blood samples, corresponding to 46 herds located in the six administrative areas were taken during 2014–2016.

#### Pigs

The estimated number of domestic swine in continental CR is close to 435500, most of them under intensive management farms, located in the Northern Huetar and Central Pacific regions [10]. A total of 2256 pigs from eight herds were sampled from 2014–2016. In addition, 160 blood samples collected at the slaughter house in the Central region were also studied. As part of the control of Wildlife Service of National Parks of CR, 58 feral pigs were sampled in the East side of Cocos Island National Park (23.85 km^2^) located in the West Pacific Ocean (5°31′08″N 87°04′18″O), during 1998–2000. This region included close to half of the area. The sampling spots were chosen randomly and their location estimated on the basis of recognized pathways and reference points already established in maps used by the National Park rangers. Ages were estimated on the basis of size, weight, secondary sexual organ development, hair distribution, hoof size and dentition. Samples were analyzed at the CR-NAHS Laboratory or at the Veterinary Medicine School, National University, Heredia, CR.

#### Horses

The estimated population of horses in CR is close to 67000 in about 20000 farms [10]. In CR there is little tradition for eating horse meat. Therefore, most of the equines are devoted to sports, recreation and work. A total of 1270 horse blood samples from 215 farms located in the six administrative areas were taken during 2014–2016.

#### Cetaceans

Thirty cetacean species have been documented in Costa Rican waters, representing about 36% of the 83 species known worldwide [11]. From 2004–2016, 115 individuals from sixteen species were reported stranded in the Costa Rican shorelines (Table 2). Cetacean blood samples were taken at the stranding sites. After death, the animals were transported to the Veterinary School of the National University of CR, for necropsy and bacteriological studies.

**Table 2:** Number of cetaceans stranded in Costa Rica from January 2004 to September 2016.

Common name	Specie	Number of animals
Striped dolphin	*Stenella coeruleoalba*	51
Bottlenose dolphin	*Tursiops truncatus*	10
Spotted dolphin	*Stenella attenuata*	8
Humpback whale	*Megaptera novaengliae*	8
False killer whale	*Pseudorca crassidens*	6
Spinner dolphin	*Stenella longirostris*	4
Rough tooth dolphin	*Steno bredanensis*	4
Dwarf sperm whale	*Kogia sima*	4
Cuvier beaked whale	*Ziphius cavirostris*	3
Risso’s dolphin	*Grampus griseus*	2
Pilot whale	*Globicephala macrorhynchus*	2
Sperm whale	*Physeter machrocephalus*	2
Common dolphin	*Delphinus delphis*	1
Beaked whale	*Mesoplodon* spp.	1
Beaked whale	*Mesoplodon* spp.	1
Sei Whale	*Balaenoptera borealis*	1
Unknown species*	Unknown	7
**Total**		**115**

#### Humans

Brucellosis in humans has been documented in CR since 1915 [3,4]. A survey for human brucellosis from 2003–2016 was carried out at the laboratories of Public Health Services (CCSS) of CR. In addition, a total of 250 abattoir workers were monitored for antibodies against *Brucella* antigens, from 2015–2016. All human case reports and bacteriology were received at the National Reference Bacteriology Laboratory at the Costa Rican Institute for Research and Training in Nutrition and Health (INCIENSA), for confirmation.

### Information collected and blood animal samples

Relevant data concerning geographical localization, size of the farm, management and characteristics of the herds or individual animals were collected. The information also included veterinary services, reproductive parameters, history of abortion/stillbirth and the presence of other domestic and wildlife species in the farms. Breeds and identifications were registered.

Blood samples were collected with syringes or a sterile vacutainers with Z serum clot activator (Vacutainer System, Greiner Bio-one), transported under refrigeration, and sera obtained by centrifugation. Each sample received a consecutive number. Analyses of the sera were performed within 24–72 hours after collection at the CR-NAHS Brucellosis Serology Laboratory or at the Immunology Laboratory at the School of Veterinary Medicine, National University, Heredia, CR. Humans blood samples were sent to the National Reference Bacteriology Laboratory (INCIENSA) for confirmation.

### Serological tests

Rose Bengal test (RBT) (ID-VET, France), indirect protein A/G ELISA (iELISA) (ID-VET, France) and competitive ELISA (cELISA) (Svanovir, SVANOVA, Sweden) and fluorescent polarization assay (FPA) (Sentry 100 instrument, Diachemix, United States) were used as diagnostic tools, as described elsewhere [12–14]. For the standardization of small ruminant brucellosis diagnostic tests, positive and negative sera from sheep and goats were obtained from Spain and Mexico respectively. Twenty sera from *B*. *melitensis* biotype 1 culture positive sheep, twenty sera from *B*. *melitensis* biotype 1 culture positive goats, twenty- one sera from non-vaccinated negative sheep and twenty-one sera from non-vaccinated negative goats were obtained and used for validation as previously described [14,15]. In Costa Rica sheep and goats are not vaccinated. Therefore, the specificity of RBT in the absence of vaccination has been estimated to be ~100%; likewise, under these conditions the sensitivity has also been estimated in ~100% [14]. The cut off values for iELISA, cELISA and FPA in sheep and goats were 120% S/P, 30% positivity and 20 milipolarization units, respectively. Since standardized diagnostic tests for water buffalo brucellosis are not available, RBT, iELISA and cELISA, including the cut-off values, were used as reported for cattle [16]. Dolphin sera were collected and tested in RBT, iELISA and cELISA as described before [17]. For swine, modified RBT, iELISA and cELISA was used as described elsewhere [18]. Likewise, for horses, background levels for the same tests were estimated with sera from 20 healthy horses with no signs of brucellosis and with no contact with cattle or small ruminants. All animal sera samples were initially screened by RBT and then by iELISA, cELISA and FPA, following the procedures described elsewhere [13,15,17]. For humans, RBT and microagglutination in 96/well round bottom plates were used for screening, as described before [19].

### Culture conditions and *Brucella* identification

Bacteriological cultures and identification of *Brucella* isolates were performed as described in the accompanying paper [2]. Briefly, various reference *Brucella* species were used as positive controls for genetic and bacteriological identification of samples [2]. According to the National Brucellosis Control Program of the CR-NAHS, seropositive sheep, goats, buffalos or pigs are selected for obligatory culling and pathological examination [20]. Necropsies were carried out at the Pathology Department in the Veterinary School of the National University, CR. Animal samples, included milk and other secretions such as vaginal swabs, semen and cerebrospinal fluid. Tissues samples included reproductive organs lymph nodes, spleen, kidney, liver and brain. In some cases aborted fetuses were also collected and sampled. Cultures were performed at the CR-NAHS or at the Bacteriology Laboratory of the Veterinary School. Non-selective and selective media, including blood agar and Columbia agar, supplemented with 5% of dextrose and sheep blood as well as Modified *Brucella* Selective Supplement Oxoid® (SR0209) and CITA medium, under 10% CO2 atmosphere, were used [21]. The selected bacterial colonies were subjected to Gram staining, agglutination with acriflavine and acridine orange dyes, tested for urease and oxidase activity, citrate utilization, nitrate reduction, H_2_S production, growth in the presence of CO_2_, thionin (20 μg/mL) and basic fuchsin (20 μg/mL) and uptake of crystal violet, according to described procedures [12].

*Brucella* DNA samples from each isolate and control strains were extracted with DNeasy Blood & Tissue kit from QIAGEN, and stored at -80°C until used. Identification of *Brucella* species was performed by bruce-ladder, single-nucleotide polymorphisms and MLVA16 analysis following standard procedures [22–25]. *Brucella* control strains were used for validation. The profiles were analyzed following standardized procedures (http://mlva.u-psud.fr/brucella/) and thereafter entered in the database MLVA-NET (http://microbesgenotyping.i2bc.paris-saclay.fr/).

### Ethical considerations

Sampling of domestic and wildlife animals is part of the National Brucellosis Control Program of the CR-NAHS [20] and the Law of Reportable Infectious Diseases of the Ministry of Health of CR [26]. Dolphin serum samples were taken from stranded dolphins following the procedures described before [27]. Protocols for the use of animal serum samples were revised and approved by the ‘‘Comité Institucional para el Cuido y Uso de los Animales de la Universidad de CR” (CICUA 057–16366), and ‘‘Comité Institucional para el Cuido y Uso de los Animales” of the National University, Heredia, CR (SIA 0545–15), and in agreement with the corresponding law ‘‘Ley de Bienestar de los Animales”, CR (Ley 7451 on Animal Welfare), and according to the “International Convention for the Protection of Animals” endorsed by Costa Rican Veterinary General Law on the CR-NAHS (Ley 8495).

Human samples were handled by the authorities of the Public Health Service of CR (Social Security Services CCSS and Ministry of Health) and then submitted to National Reference Bacteriology Laboratory at INCIENSA for diagnostic confirmation. In this institution the samples were handled according to the INCIENSA ethical committee specifications and the agreement between INCIENSA and SENASA (Oficio 16-06-2013). Upon registration to the Medical Health Centre, all patients were informed regarding the purpose of the work and provided the corresponding written consents according to the respective Law (Ley 9234, La Gaceta 79). All samples were taken following the procedures dictated by the Costa Rican National Health system (Ley 9234, La Gaceta 79), and the World Medical Association Declaration of Helsinki (Ethical Principles for Medical Research Involving Human Subjects, General Assembly, Seoul, October 2008), regarding blood samples.

### Statistics

For sheep and goats, the sample sizes were determined according to Cannon and Roe [28] using Win Episcope 2.0 software [29], with an expected brucellosis prevalence of 0.6% for sheep and 0.7% for goats, with a confidence level of 95%. This estimation included 500 sheep and 413 goats to be sampled, distributed in 10 and 13 herds respectively, sorted by region as described above. Herd selection was chosen assuming that the management and biosecurity actions, regarding these two ruminants, are similar in CR. Herds were chosen randomly from sections “A” and “B”, which are the broodstock herds, and largely reflected the sanitary conditions of section “C”. From each herd selected, a proportional sample population was calculated based on the clinical signs compatible with brucellosis, with a confident level of 95% and an expected prevalence of 5%, according with Cannon and Roe [28]. In addition to the random sampling, and in order to increase the probability of positive results, a biased priority was given to females with a history of abortions, weak or stillborn births, placenta retention, or with conditions that rendered individuals more susceptible to any infection, such as low body condition and pale mucous membranes. If the total number of animals defined for the herd was not covered with these specifications, random adult females were selected. Breeding rams in each farm were also examined for the detection of orchitis, epididymitis and reproductive problems. For feral pigs, the size of the sample was selected for an expected maximum population of 500 pigs distributed in the entire island, with a 95% confidence level and a tentative prevalence of 5%. The rest of the animal species sampled corresponded to the surveillance performed as part of the National Brucellosis Control Program of the CR-NAHS and according to the OIE specifications [13].

## Results

### Sheep and goats

**Fig 1:**
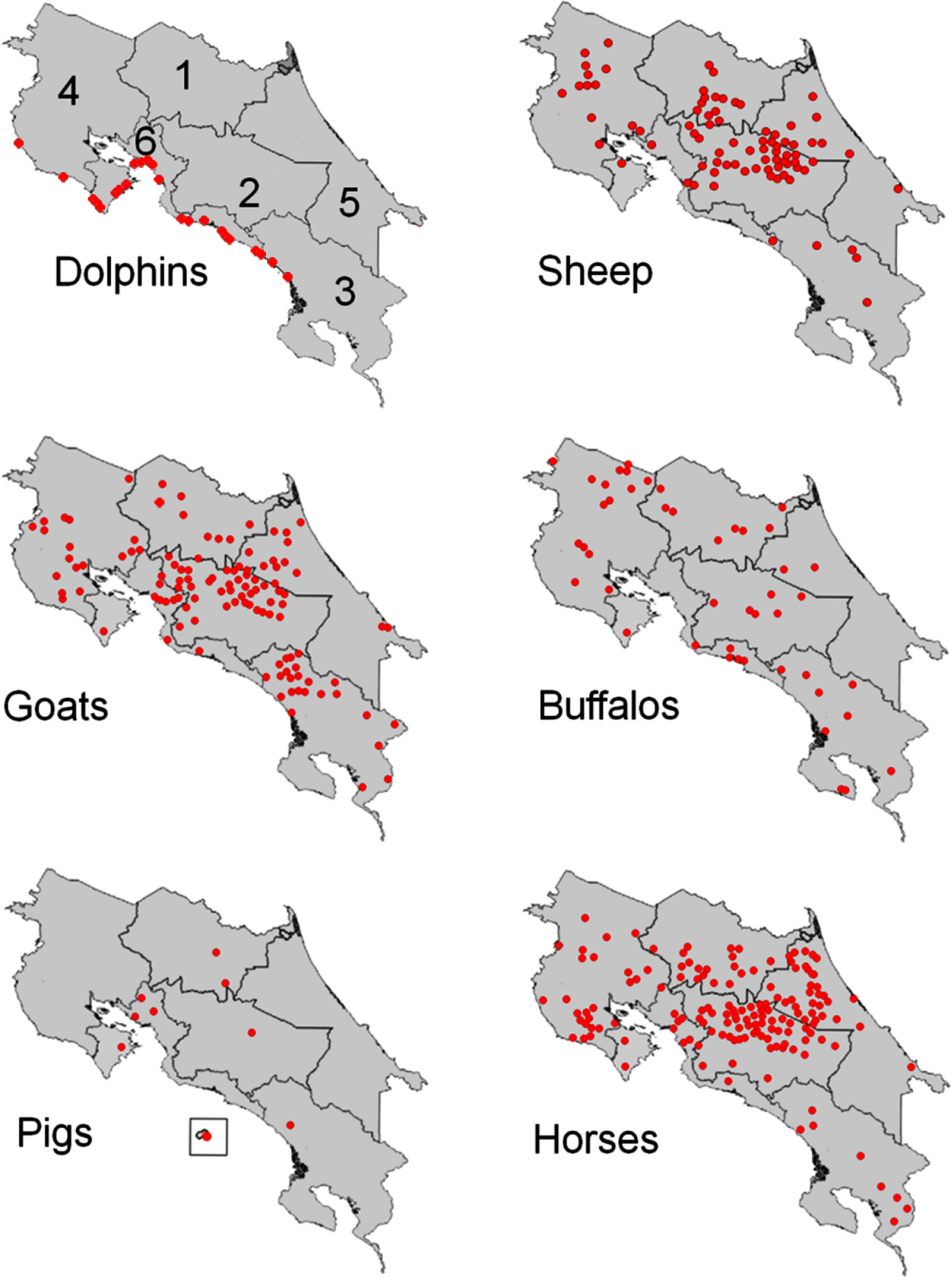
Sampling of animal stocks, in the six regions of CR. The epidemiological regions are as follow: 1, Northern Huetar; 2, Central; 3, Brunca; 4, Chorotega; 5, Caribbean Huetar; 6, Central Pacific. Each red dot represents an animal stock facility.

Most of the ovine and caprine herds are located in the lowlands of CR (below 1000 m) and are mainly devoted to dairy (caprine) and meat (ovine) production (Table 1). The sampling procedure was carried out at the indicated regions, from 2015–2016 (Fig 1). From a total of 510 sheep sampled, corresponding to 10 herds, eleven animals (five herds) were RBT positive and five cELISA positive. None of the RBT positive animals were positive in iELISA, cELISA or FPA. Likewise, from a total of 424 goats, covering close to 10% of the Costa Rican population, only five animals demonstrated positive reactions in RBT. However, none of these RBT positive samples resulted positive in iELISA, cELISA or FPA. According to these results, the estimated brucellosis RBT prevalence values for goat and sheep herds were 0.98% and 0.7%, respectively. The RBT positive animals were culled and tested for the presence of *Brucella* spp. in lymph nodes, spleen, liver, placenta, mammary gland, milk and fetus organs. All cultured samples tested negative for *Brucella* spp. Epidemiological and clinical surveys of the sheep and goat populations and the corresponding farms did not demonstrate clinical brucellosis.

From the 3681 ovine and caprine routinely sampled at the CR-NAHS laboratories for regular diagnosis, only one caprine was classified as positive in RBT and iELISA. The animal was slaughter and their various organs tested for the presence of *Brucella*, with negative results. Clinical disease compatible with *B*. *ovis* infection was not detected in rams. Likewise, this bacterium was not isolated from semen samples. Taken together these data, the “positive” RBT reactions were estimated as unspecific and the presence of brucellosis in ovine and caprine herds ruled out.

### Water buffalos

Most water buffalos are located in the low lands, since they require fresh water habitats for subsistence. From a total of 2586 samples distributed in 46 herds, collected from 2014–2016 (Fig 1), 17 animals tested positive in RBT, 38 in cELISA and 77 in the iELISA. The total number of herds positive in these three assays was ten. All RBT positive samples were also positive in iELISA and cELISA; and all samples positive for cELISA were also positive in iELISA. FPA was not performed. In spite of the efforts, *Brucella* organisms were not isolated from vaginal swabs, dairy products, placental tissues, fetuses, testes, lymph nodes, mammary gland, blood, spleen or liver of the culled seropositive animals. However, due to the reported clinical characteristics and the testimonies of persistent abortions and positive serological reactions, *Brucella* infections were suspected. Moreover, it is likely that *B*. *abortus* constitutes an infection source for water buffaloes, since bovine brucellosis caused by this *Brucella* specie is highly prevalent in CR [2].

### Pigs

From the number of herds studied and the samples obtained at the slaughter house (Fig 1), only two pigs of one herd were RBT positive. From these, only one pig was also positive in iELISA and cELISA. The FPA assay was not performed. Positive animals were culled and different tissues were cultured for the presence of *Brucella*, with negative results. In addition, tissues of aborted fetuses in some farms were also tested for the presence of *Brucella*, all with negative results. Likewise, positive serological reactions were not detected in the feral pig population in the Cocos Island. Histopathological examination of the liver in the feral swine sample showed chronic inflammation in 84% of the cases, while 20% had multifocal granulomatous inflammation with eosinophilic infiltration, probably related to the presence of parasite nematode *Stephanurus dentatus*, but not *Brucella*. Taken together these data, the positive serological reactions were estimated as non-specific.

### Horses

Most horses are located in North Huetar, Chorotega and the northern part of the Caribbean Huetar regions of CR. Therefore, most of the samples are from these areas (Fig 1). From the total number of farms studied 14 (6.5%) had seropositive animals, including 18 horses positive in RBT; from these, only four were also positive in both iELISA and cELISA. In spite of the efforts, *Brucella* was not isolated from horses. However, it is likely that *B*. *abortus* is a source of infection in horses, since many of these animals are in close contact with infected bovines in CR. In addition, some clinical features such as fistulous withers and nonspecific lameness due to joint infection, have occasionally been observed in horses.

### Cetaceans

Cetacean brucellosis in Costa Rican was investigated from 2004–2016. RBT and iELISA, designed for cetacean diagnosis, were positive in 54 (46.9%) individuals from six different species. They included 38 striped dolphins (*Stenella coeruleoalba*), one bottlenose dolphins (*Tursiops truncatus*), one spotted dolphins (*S*. *attenuata*), one common dolphin (*Delphinus delphis*), one rough toothed dolphin (*Steno bredanensis*), and one Cuvier beaked whale (*Ziphius cavirostris*). However, striped dolphin (*S*. *coeruleoalba)* remains as the only cetacean specie from which *B*. *ceti* has been isolated from different organs in CR.

Strong positive RBT and iELISA reactions were obtained in sera from 37 out of 38 striped dolphins stranded at the Pacific coast of CR (Fig 1). Thirty-seven out of 38 striped dolphins, stranded alive. At the time of stranding, all live animals presented neurological symptoms such as tremors, buoyancy difficulties, weakness, seizures and locomotion problems. With exception of two dolphins (one seropositive and one seronegative), all other *S*. *coeruleoalba* dolphins displayed neurobrucellosis, following previous diagnosis [27]. All of them died at the stranding site within hours after the event. Necropsy was performed in all cases and *B*. *ceti* was isolated from the cerebrospinal fluid of 29 individuals (70%). In addition, *B*. *ceti* was also present in placenta, umbilical cord, amniotic and allantoic fluids, multiple fetal organs, milk, cardiac valve, atlanto occipital joint fluid, lung and lung nematodes (*Halocercus spp*.) [6,27,30,31]. All *B*. *ceti* isolates belonged to the MLVA16 type P [32], corresponding to the Pacific Ocean (data accessible at: http://microbesgenotyping.i2bc.paris-saclay.fr/ [and the following entries: public databases, Brucella v4_1, bmarCR+number, years 2006–2014]).

### Humans

**Fig 2:**
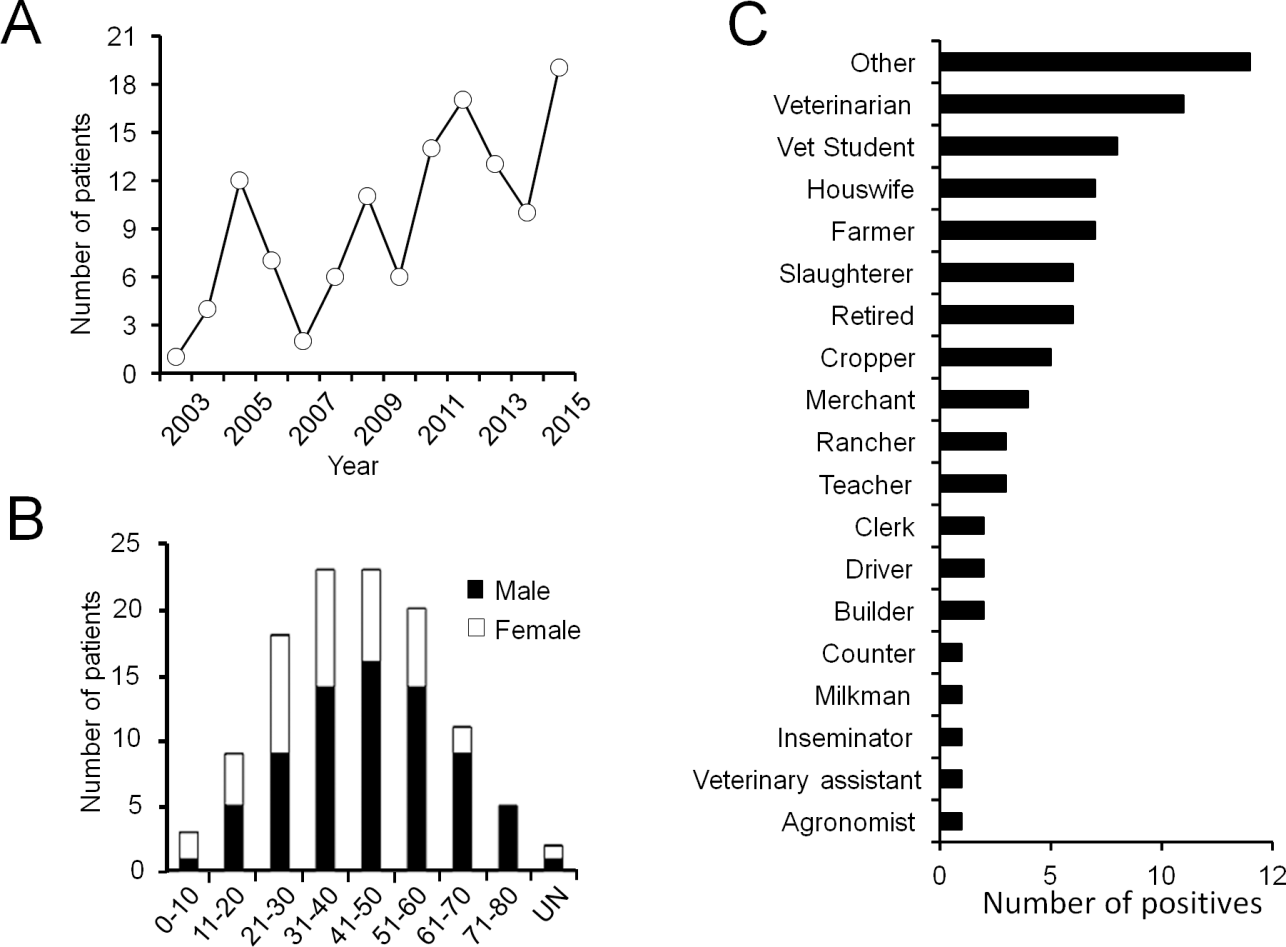
Occurrence human brucellosis cases in CR from 2003–2015. (A) Number of human brucellosis cases diagnosed per year in CR for the period. All cases recorded were due to *B*. *abortus*. (B) Distribution per age and proportion of male and female brucellosis cases in CR, diagnosed for the period. (C) Proportion of 250 seropositive abattoir workers from 2015 to 2016.

According with the Costa Rican National Reference Bacteriology Laboratory (INCIENSA), the number of human cases reported by the health centers over 12 year (2003–2015) period corresponded to 124 patients (Fig 2A): fifty one were from the Central region 37 from the Caribbean Huetar region and 36 cases from all other regions. Male and female patients represented 79 and 41 cases (Fig 2B), respectively, with ages ranging between 8–76 year-old, with a large proportion of veterinarians, farmers and slaughter plant workers (Fig 2C). From a total of the 250 abattoir workers only three presented high antibody titers (>1/160) compatible with an active brucellosis. With the exception of two *B*. *neotomae* isolates [7], all other human brucellosis cases corresponded to *B*. *abortus*.

## Discussion

For most of the history of CR, sheep and goats have been raised in very low numbers and the dairy products and meat of these animals barely consumed [33]. Until 1975 the number of goats and sheep in the country were close to 1000 animals, all together [33]. However, in the nineties the population of these small ruminants started to increase. Already, in the first decade of the XXI century, the numbers of goats and sheep were close to 5000 and 3000, respectively [34]. With the enhanced acceptance of ovine and caprine dairy and meat products, the emergent industries for small ruminants have increased. Indeed, the numbers of goats and sheep have augmented almost three fold (12852) and twelve fold (35800) [10], respectively.

An epidemiological survey for caprine and ovine brucellosis was performed from 2015–2016. Although we detected a minor number of RBT positive reactions in small ruminants, they were regarded as false positives. In spite of the high specificity and sensitivity displayed by the RBT under controlled conditions with a limited number of known sera, this assay is not perfect and some non-specific reactions are expected to occur under field conditions. Therefore, exhaustive clinical, pathological and epidemiological investigations in the serologically positive sheep and goats were carried out, all rendering negative results for the presence of *Brucella* infections. Bacteria displaying similar antigenic determinants as smooth brucellae may be the source of false positive reactions [35]. In addition, positive serological reactions due to *B*. *abortus* infections cannot be ruled out, since this bacterium is highly prevalent in CR [2]. However, we did not isolate *B*, *abortus* or any other brucellae from the tissues of goats and sheep. Although *B*. *melitensis* may be present in some Central American countries [36], this bacterium has never been isolated in animals or humans in CR [5, 36]. Following this, it is important to keep these small ruminants free of brucellosis, restricting the importation of animals and semen from *B*. *melitensis* free countries.

Similar to goats and sheep, the number of water buffalos has steadily increased in CR during the last ten years. In 2006 the number of water buffalos in CR was close to 615 animals [37]; in ten years the population has increased twenty fold, most of them devoted to the production of dairy products. Taking into account the persistent positive serological reactions, their close association of water buffalo with *B*. *abortus* infected cattle and the reported cases of abortions compatible with clinical disease; we believe that some water buffalo populations are infected with *Brucella* in CR. Moreover, a significant number of the CR water buffalo population originates from Trinidad-Tobago, country endemic for water buffalo brucellosis [8, 38]. The fact that we did not isolate *Brucella* from water buffalos may be related to the natural resistance of these animals to brucellosis in relation to other bovines [39].

*B*. *suis* was isolated from a domestic pig in the Central region of CR in 1984 [5]. Since then, the bacterium has not been isolated from boars, in spite of the efforts. In CR pigs seldom roam freely around the houses and most animals are confined to intensive management facilities, under good health conditions. Moreover, with the exception of Cocos Island, no feral pigs are present in the CR territory. Since no clinical or epidemiological surveys indicate swine brucellosis, it is unlikely that *B*. *suis* is currently infecting pigs in the country.

Horses are not primary *Brucella* hosts and commonly they do not have the ability to transmit the bacterium to other animals or humans. Therefore, horses are not of epidemiological relevance in keeping the bacterium life cycle; however, these animals are sentinels for the presence of *Brucella* in other animals, mainly in cattle. Like humans, they become infected by contact with abortions or with infected cattle, and display a wide range of clinical manifestations including articular swelling and general weakness [40]. The fact that close to 18 horses displayed recurrent positive reactions against *Brucella*, may be an indication of the high seroprevalence of *Brucella* infections in cattle [2], including water buffalo.

*B*. *ceti* infections in dolphins stranded in the CR Pacific coast were detected for the first time in 2004 [6]. A total of 115 stranding events from at least 16 different species of cetaceans have been recorded in CR seashores from 2004–2016 (Table 2). From these, six species displayed positive serological reactions. However, *B*. *ceti* active infections have been only documented in striped dolphins from the Pacific Ocean of CR. All *B*. *ceti* isolates belong to the same MLVA16 type P. This bacterial group corresponds to a particular cluster distinct from other *B*. *ceti* strains isolated in various oceanic latitudes, and it is a hallmark for *S*. *coeruleoalba* infections in the Eastern Tropical Pacific [32]. Moreover, all the 29 dolphin cases in which *B*. *ceti* organisms were isolated suffered from neurobrucellosis [27]. It seems, therefore, that this dolphin specie is highly susceptible to *B*. *ceti* and that many of the stranding events were due to brain infections, as recorded in other latitudes [41]. The surveillance of cetacean brucellosis in Central American littorals requires attention. This is mandatory to understand the impact that brucellosis has in the Eastern Tropical Pacific marine mammal populations and to ensure prevention measures for potential human and animal infections [42].

In a previous study in the Central region (Cartago, CR), in which 71% of the human population consumed unpasteurized dairy products; an overall seroprevalence of 0.87% was detected [19]. However, no statistically significant association was found between unpasteurized milk consumption and the presence of antibodies against *Brucella* organisms. Here, we reported a steady increase in the number of human brucellosis cases during a lapse of 12 years. Whether the steady increase of human brucellosis reports corresponded to improved diagnosis or to intensification in the number of cases, is not known. The number of human brucellosis cases due to *B*. *abortus* is consistent with the high prevalence of bovine brucellosis in CR, and the absence of *B*. *melitensis* in sheep and goats, and *B*. *suis* in pigs, two *Brucella* species that display a higher zoonotic potential than former bacteria [43]. In CR there are other zoonotic brucellae such as *B*. *neotomae* [7] and *B*. *canis* [44], which were not considered in this study. Nevertheless, a careful identification of strains is required, even with those *Brucella* species that are considered of low zoonotic risk.

From the epidemiological perspective, it seems that the population of sheep, goats and pigs in CR are free of *B*. *melitensis* infections. This seems to be also the case for *B*. *ovis* in rams and *B*. *suis* for pigs. Consequently, humans are also free of these bacterial species. However, with the increasing number of small ruminant species in the country the risk of *Brucella* infections arriving from other latitudes requires permanent surveillance, improved management and sensitive and specific diagnostic tools.

## Conclusions

1. Domestic ovine, caprine and swine herds are free of brucellosis in CR.
2. The presence of *Brucella* infections in water buffaloes is highly suspected in CR.
3. The presence of *B*. *abortus* infections in horses is highly suspected in CR.
4. Striped dolphins from the Pacific Ocean of CR are the main host of *B*. *ceti* cluster type P.
5. The main clinical symptom found in striped dolphins corresponded to neurobrucellosis.
6. Detection of human infections, due to *B*. *abortus*, has steadily increased since 2005 in CR.
7. Estimating the presence of *Brucella* infections in different hosts inhabiting CR is relevant for understanding the impact that brucellosis has in the country and for prevention measures.
